# Supplementary material for: Antimicrobial Resistance Profiles and Macrolide Resistance Mechanisms of Campylobacter coli Isolated from Pigs and Chickens
Source: Microorganisms. 2021 May 17;9(5):1077. doi: 10.3390/microorganisms9051077 (PMC8156767; doi:10.3390/microorganisms9051077)
Supplement: Supplementary file 1 [file microorganisms-09-01077-s001.zip › microorganisms-1230495-supplementary.pdf]

Table S1. The number of *Campylobacter coli* isolates recovered from fecal and carcass samples of apparently healthy and diseased pig and chicken between 2010 and 2118 in Korea.

Year of isolation	Number of isolates					
	Pig			Chicken		
	Feces	Carcass	Subtotal	Feces	Carcass	Subtotal
2010	46	12	58	38	25	63
2011	47	54	101	6	29	35
2012	37	64	109	43	55	98
2013	9	36	45	0	7	7
2014	62	75	137	27	19	46
2015	12	69	81	7	43	50
2016	11	24	35	32	76	108
2017	9	34	43	28	44	72
2018	25	9	34	46	50	96
Total	266	377	643	227	348	575

Table S2. Antimicrobial resistance patterns in *C. coli* isolated from pigs and chickens from 2010 to 2018 in Korea.

Number of Antimicrobials	Resistance patterns	Number of patterns
	**Pig isolates (*n*=643)**	
0		0
1	**TET (n=23)**, TEL(*n*=1), NAL (*n*=1), GEN (*n*=1), CIP (*n*=1)	**5**
2	**CIP NAL (*n*=56)**, CIP TET (*n*=4), CLI TET (*n*=3), CIP ERY (*n*=1), CLI TEL (*n*=1), GEN TET (*n*=1), TEL TET (*n*=1)	7
3	**CIP NAL TET (*n*=187)**, CIP NAL TEL (*n*=18), CIP FFC NAL (*n*=6), AZM CIP NAL (*n*=2), IP GEN NAL (*n*=1), CIP CLI NAL (*n*=1), CLI FFC TET (*n*=1), AZM ERY TET (*n*=1)	8
4	**CIP GEN NAL TET (*n*=14)**, CIP NAL TEL TET (*n*=10), CIP FFC NAL TET (*n*=9), AZM CLI ERY TET (*n*=6), CIP CLI NAL TET (*n*=5), AZM CLI ERY TEL (*n*=5), CIP GEN NAL TEL (*n*=3), CIP CLI NAL TEL (*n*=2), CIP CLI FFC NAL (*n*=1), AZM CIP NAL TET (*n*=1)	10
5	**CIP CLI FFC NAL TET (*n*=13),** AZM CLI ERY TEL TET (*n*=12), CIP FFC GEN NAL TET (*n*=4), CIP CLI NAL TEL TET (*n*=3), AZM CIP CLI ERY NAL (*n*=2), AZM CIP ERY NAL TET (*n*=2), CIP GEN NAL TEL TET(*n*=2), AZM CIP ERY GEN NAL (*n*=1), CIP FFC NAL TEL TET (*n*=1),	9
6	**AZM CIP CLI ERY NAL TEL (*n*=16)**, AZM CIP CLI ERY NAL TET (*n*=9), AZM CLI ERY FFC TEL TET (*n*=4), AZM CIP ERY GEN NAL TET (*n*=2), AZM CIP CLI ERY GEN NAL (*n*=1), AZM CIP CLI ERY TEL TET (*n*=1), AZM CIP CLI GEN NAL TEL (*n*=1), AZM CIP CLI GEN NAL TET (*n*=1), AZMCIP ERY GEN NAL TEL (*n*=1), AZM CLI ERY GEN TEL TET (*n*=1), CIP CLI FFC GEN NAL TET (*n*=1), CIP CLI FFC NAL TEL TET (*n*=1)	12
7	**AZM CIP CLI ERY NAL TEL TET (*n*=131)**, AZM CIP CLI ERY NAL TEL TET (*n*=6), AZM CIP CLI ERY NAL TEL TET (*n*=1)	3
8	**AZM CIP CLI ERY GEN NAL TEL TET (*n*=37)**, AZM CIP CLI ERY GEN NAL TEL TET (*n*=7)	2
9	**AZM CIP CLI ERY GEN NAL TEL TET (*n*=5)**	1
Total		57
	**Chicken isolates (*n*=575)**	
0		0
1	**CIP (*n*=2**)	1
2	**CIP NAL (*n*=94)**, AZM NAL (*n*=1), GEN TET (*n*=1)	3
3	**CIP NAL TET (*n*=269)**, CIP GEN NAL (*n*=20), CIP GEN TET (*n*=1)	3
4	**CIP GEN NAL TET (*n*=79)**, CIP NAL TEL TET (*n*=5), CIP CLI NAL TET (*n*=3), CIP FFC NAL TET (*n*=2), CIP CLI FFC NAL (*n*=2), AZM CIP NAL TET (*n*=1),	6
5	**AZM CIP CLI ERY NAL (*n*=1)**, CIP FFC GEN NAL TET (*n*=1), AZM CIP GEN NAL TET (*n*=1)	3
6	**AZM CIP CLI ERY NAL TET (*n*=12)**, AZM CIP CLI ERY NAL TEL (*n*=9), AZM CLI ERY GEN NAL TET (*n*=1), CIP CLI GEN NAL TEL TET (*n*=1)	4
7	**AZM CIP CLI ERY NAL TEL TET (*n*=42)**, AZM CIP CLI ERY GEN NAL TET (*n*=2), AZM CIP CLI ERY FFC NAL TEL (*n*=1), AZM CIP CLI ERY NAL TEL TET (*n*=1)	4
8	**AZM CIP CLI ERY GEN NAL TEL TET (*n*=15)**, AZM CIP CLI ERY FFC NAL TEL TET (*n*=1)	2
9	**AZM CIP CLI ERY FFC GEN NAL TEL TET (*n*=3)**	1
Total		27

Abbreviations: AZM, azithromycin; CIP, ciprofloxacin; clindamycin, CLI; ERY, erythromycin; FFC, florfenicol; GEN, gentamicin; NAL, nalidixic acid; TEL, telithromycin; TET, tetracycline
